# 591 The Use of Opioids in Adult Burn Patients: Long-term Dependency Risk and Outcomes

**DOI:** 10.1093/jbcr/iraf019.220

**Published:** 2025-04-01

**Authors:** Julia Kleinhapl, Jaclyn Dempsey, Juquan Song, Oscar Suman, Ludwik Branski, Steven Wolf

**Affiliations:** University of Texas Medical Branch; University of Texas Medical Branch; University of Texas Medical Branch; University of Texas Medical Branch; University of Texas Medical Branch; University of Texas Medical Branch

## Abstract

**Introduction:**

Pain management in burn care remains a challenge in both acute and long-term treatment. Among non-steroidal anti-inflammatory drugs (NSAIDs), acetaminophen, gabapentinoids, and opioids are widely used as systemic analgesics, especially in the acute setting. However, due to a high potential for dependency and notable adverse effects, duration of treatment should be short. In this study, we investigated the impact of opioid administration after burns on dependency risk and outcomes in a large database.

**Methods:**

We conducted a study in the real-world database TriNetX. Patients were stratisfied into cohorts based on the affected total body surface area (TBSA): Cohort 1 (< 20% TBSA burned), Cohort 2 (20-39% TBSA burned), Cohort 3 (>40% TBSA burned). We analysed treatment with opioids within 3 months after burn, assessed the frequency of opioid related disorders (dependence, abuse, intoxication) within 3 years after burn, and compared outcomes in treated vs. untreated patients. Statistical analysis was performed with significance set at p< 0.05.

**Results:**

In those < 20% TBSA burned, 51% (92,446/182,113) were treated with opioids, while 61% (3,389/5,557) and 52% (2,230/4,249) were treated in cohorts 2 and 3, respectively. Opioid related disorders occurred in 2.2% (2,149/92,446) in cohort 1, 1.8% (62/3,389) in cohort 2 and 3.3% (74/2,230) in cohort 3. Cohort 1 had significantly higher risk for mortality, skin infections, wound healing disorders, pneumonia, sepsis, renal failure, anemia and psychological impairment (p< 0.0001 each) in opioid treated patients. Those with TBSA burned between 20-39% showed a significantly higher risk for mortality and anemia (p< 0.0001 each) when being treated, while untreated patients showed a significantly higher risk for pneumonia (p< 0.0001) and renal failure (p=0.0004). In cohort 3, those receiving opioid treatment had a lower incidence of sepsis (p< 0.0308), pneumonia (p=0.0050) and renal failure (p< 0.0001), but a significantly higher incidence of skin infections (p=0.0005), anemia (p=0.0001) and mortality (p< 0.0001).

**Conclusions:**

While opioids are commonly used, these have a reasonably low incidence of dependence (< 4%) but are associated with higher risk for adverse events, especially in those with less severe burns. In lower TBSA cohorts, opioid administration is associated with increased mortality, while severely burn injured patients profit from the use of opioids, showing reduced incidence of complications such as sepsis or pneumonia.

**Applicability of Research to Practice:**

Our findings highlight the need for ongoing research to optimize pain management strategies, maximize therapeutic outcomes, and minimize complications.

**Funding for the Study:**

Remember the 15 Endowment. Database funding by the National Center for Advancing Translational Sciences.

